# Telmisartan ameliorates insulin sensitivity by activating the AMPK/SIRT1 pathway in skeletal muscle of obese *db/db* mice

**DOI:** 10.1186/1475-2840-11-139

**Published:** 2012-11-08

**Authors:** Asuka Shiota, Michio Shimabukuro, Daiju Fukuda, Takeshi Soeki, Hiromi Sato, Etsuko Uematsu, Yoichiro Hirata, Hirotsugu Kurobe, Norikazu Maeda, Hiroshi Sakaue, Hiroaki Masuzaki, Iichiro Shimomura, Masataka Sata

**Affiliations:** 1 Department of Cardio-Diabetes Medicine, University of Tokushima Graduate School of Health Biosciences, 3-18-15 Kuramoto, Tokushima, 770-8503, Japan; 2 Department of Cardiovascular Medicine, University of Tokushima Graduate School of Health Biosciences, Tokushima, Japan; 3 Department of Nutrition and Metabolism, University of Tokushima Graduate School of Health Biosciences, Tokushima, Japan; 4 Department of Cardiovascular Surgery, University of Tokushima Graduate School of Health Biosciences, Tokushima, Japan; 5 Department of Metabolic Medicine, Osaka University Graduate School of Medicine, Osaka, Japan; 6 Division of Endocrinology, Diabetes and Metabolism, Hematology, Rheumatology, Second Department of Internal Medicine, University of the Ryukyus, Graduate School of Medicine, Okinawa, Japan

**Keywords:** Adiponectin, AMP-activated protein kinase, Obesity, Peroxisome proliferator-activated receptor-γ, SIRT1

## Abstract

**Background:**

Telmisartan is a well-established angiotensin II type 1 receptor blocker that improves insulin sensitivity in animal models of obesity and insulin resistance, as well as in humans. Telmisartan has been reported to function as a partial agonist of the peroxisome proliferator-activated receptor (PPAR) γ, which is also targeted by the nicotinamide adenine dinucleotide (NAD)-dependent deacetylase (SIRT1). Here, we investigated the pathways through which telmisartan acts on skeletal muscle, *in vitro* as well as *in vivo*.

**Methods:**

Nine-week-old male *db/db* mice were fed a 60% high-fat diet, with orally administrated either vehicle (carboxymethyl-cellulose, CMC), 5 mg/kg telmisartan, or 5 mg/kg telmisartan and 1 mg/kg GW9662, a selective irreversible antagonist of PPARγ, for 5 weeks. Effects of telmisartan on *Sirt1* mRNA, AMPK phosphorylation, and NAD+/NADH ratio were determined in C2C12 cultured myocytes.

**Results and discussion:**

Telmisartan treatment improved insulin sensitivity in obese *db/db* mice fed a high-fat diet and led to reduction in the size of hypertrophic pancreatic islets in these mice. Moreover, *in vitro* treatment with telmisartan led to increased expression of *Sirt1* mRNA in C2C12 skeletal muscle cells; the increase in Sirt1 mRNA in telmisartan-treated C2C12 myoblasts occurred concomitantly with an increase in AMPK phosphorylation, an increase in NAD+/NADH ratio, and increases in the mRNA levels of PGC1α, FATP1, ACO, and GLUT4.

**Conclusions:**

Our results indicate that telmisartan acts through a PPARγ-independent pathway, but at least partially exerts its effects by acting directly on skeletal muscle AMPK/SIRT1 pathways.

## Introduction

The nicotinamide adenine dinucleotide (NAD)-dependent deacetylase (SIRT1) has been reported to be involved in protection against metabolic disorders as well as in enhancing life span [1]. Evidence suggests that SIRT1 also plays a fundamental role in the metabolism and differentiation of skeletal muscle cells [2], and transgenic or knockout *Sirt1* mouse models have implicated the protein in protection of skeletal muscle against oxidative stress [3,4]. It has been speculated that SIRT1 plays an important role in the regulation of transcriptional networks in various critical metabolic processes [1]. The SIRT1 signal seems to be mediated by an increase in fatty acid oxidation by activation of AMP-activated protein kinase (AMPK) in skeletal muscle [5]. The AMPK activators metformin [6] and A-769662 [7], as well as resveratrol [8], a polyphenolic SIRT1 activator, ameliorate insulin resistance in animals given a high-fat diet.

Telmisartan is a well-established angiotensin II type 1 receptor (AT1) blocker that improves insulin sensitivity in rodents that have received high-fat–containing diets [9-12], as well as in diabetic [12] and nondiabetic patients [13]. Telmisartan has been reported to function as a partial agonist of the peroxisome proliferator-activated receptor (PPAR) γ, a member of the ligand-activated nuclear receptor superfamily that is expressed at high levels in adipose tissue [14]. PPARγ regulates genes that modulate lipid utilization and storage, lipoprotein metabolism, adipocyte differentiation, and insulin action [14]. Thus, it is possible that the antidiabetic effects of telmisartan depend largely on PPARγ-dependent mechanisms [15]. PPARγ and the PPARγ coactivator (PGC)-1α, which play key roles in various metabolic disorders, are also known to be targeted by SIRT1 [16,17], but the effects of telmisartan on SIRT1- and PPARγ-signaling remain unclear.

In the present study, we investigated whether telmisartan acts on skeletal muscle through an AMPK/SIRT1 pathway in skeletal muscle.

## Materials and methods

### Animals and experimental protocol

Study protocols were approved by the Committee on Animal Research, the University of Tokushima, and have been done on the basis of ethical principles and guidelines for experiments on animals (http://freedownload.is/pdf/ethical-principles-and-guidelines-for-experiments-on-animals-22328952.html). Nine-week-old male *db/db* mice (Charles River Laboratories Japan Inc. Tokyo, Japan) were fed a high-fat diet (HFD-60, Oriental Yeast Co., Ltd., Tokyo) *ad libitum* for 5 weeks. HFD-60 contains energy content 62% fat (lard 33, milk-casein 26, corn-starch 16 g/100 g·diet; fatty acid 16:0 24.4, C18:0 13.8, C:18:1 41.8, 18:2(n-6) 12.0 g/100 g of total fatty acids), 18% protein and 20% carbohydrate, compatible with the AIN-93 G recommendations [18]. *db/db* mice were separated into 3 groups and were orally administrated either vehicle (carboxymethyl-cellulose, CMC), 5 mg/kg telmisartan, or 5 mg/kg telmisartan and 1 mg/kg GW9662 (Sigma, St. Louis, MO), a selective irreversible antagonist of PPARγ, once a day, for 5 weeks. Age-matched male wild type +/+ mice (Charles River Laboratories Japan) fed a normal diet, containing energy content 10% fat, 14% protein and 76% carbohydrate (AIN-93 M, Oriental Yeast Co. Ltd.) were used as a control group.

We defined the dose usage of telmisartan and GW9662 as follows. (1) telmisartan (5 mg/kg/day). For hypertensive patients, telmisartan is orally administered at the dose of 20‐160 mg per day, which is equivalent to ≈ 0.3-3 mg/kg/day. A pharmacokinetic study showed that steady state Cmax of telmisartan was 28.3 ng/ml at 20 mg and 592 ng/ml at 120 mg (≈ 0.06-1.15 μmol/L) in normotensive elderly subjects [19]. We had preliminary confirmed telmisartan show a PPAR-γ agonistic effect at doses of 0.5-10 μM in HEK293 cells (data not shown). Combined, we selected the dose of 5 mg/kg/day to obtain a PPAR-γ agonistic effect at a clinical dose. (2) GW9662 (1 mg/kg/day). We determined the dose of GW9662 following Goyal et al [20]. They showed that the protective effects of telmisartan (10 mg/kg/day) on myocardial infarction model in experimental diabetes could be modulated by down-regulated PPAR-γ expression by 1 mg/kg/day of GW9662 [20].

Body weight, food intake, systolic blood pressure and blood glucose were measured weekly. Blood pressure was measured using a tail-cuff system (Softron, Co., Tokyo) and glucose was determined by a glucose oxidase test. Mice were housed in a light- and temperature-controlled room, with a 12-hour light/dark cycle.

At the end of the experimental period, an insulin tolerance test (ITT) was performed. Mice were given an intraperitoneal injection of 0.75 U/kg insulin, after which blood glucose concentrations were measured at 0, 15, 30, 60, and 120 min. Moreover, at the end of the experimental period, serum, and subcutaneous and visceral fat, were collected and stored at -80°C until assayed. Insulin was determined by a mouse insulin ELISA kit (AKRIN-011 T, Shibayagi, Gunma, Japan).

Distribution of adipocyte size was determined as previously described [21]. Briefly, adipocytes isolated from visceral and subcutaneous adipose tissue were fixed with 2% osmium tetroxide and passed through a 250-μm nylon filter to remove the fibrous elements, after which the cells were washed extensively with isotonic saline. 10000 cells were analyzed using the Coulter Multisizer III (Beckman Coulter, High Wycombe, England). Paraffin-embedded serial sections of pancreata (5-μm thickness) were stained for hematoxylin and eosin (HE) and indirect insulin immunostaining [22].

### Cell culture

C2C12 myoblasts were cultured in Dulbecco’s modified Eagle’s medium (DMEM; Sigma) with 4.5 g/L glucose supplemented with 10% fetal bovine serum (FBS; Sigma), 100 U/mL penicillin, and 100 μg/mL streptomycin as previously described [23]. One day after C2C12 myoblasts reached confluence (day 0), cells were induced to differentiate into myocytes by replacing the medium with a low serum differentiation medium (2% horse serum; Gibco®, Life Technologies Japan, Ltd., Tokyo) for 2 days. After 48 h, the induction medium was removed and cells were maintained in DMEM with 10% FBS and, thereafter, the medium was changed every 2 days until day 5. Telmisartan was dissolved in dimethyl sulfoxide (DMSO) and added to culture media within 0.1% of volume (telmisartan final concentration: 0 - 1 μM). Telmisartan treatment was performed on day 5.

### NAD^+^/NADH measurements

NAD^+^ and NADH nucleotides were measured with an enzymatic NADH recycling assay, using the NAD^+^/NADH Quantification kit (BioVision, Mountain View, CA) following the manufacturer’s recommended protocol [24]. C2C12 myotubes were collected in 200 μl of NADH/NAD^+^ extraction buffer by subjecting the cells to 2 cycles of freeze/thaw. The supernatants were divided into 2 sets: to detect NADH, 1 set of supernatants was heated to 60°C for 30 min to decompose NADH, while those from the other set was used to measure the total NADH plus NAD^+^-content, by performing the cycling assay in the absence of thermal decomposition. Then, the NAD^+^/NADH ratio was calculated.

### Semi-quantitative RT-PCR analysis

Total RNA was isolated from C2C12 myotubes using RNAiso plus (Takara Bio Inc., Shiga, Japan). Complementary DNA synthesis was performed, using a QuantiTect® Reverse Transcription kit (SA Biosciences, Boston, MA), from 1 μg of the extracted total RNA. Then, we performed real-time RT-PCR with gene-specific primers and SYBR green dye using an Applied Biosystems 7500 Real-Time PCR System (Life Technologies Japan Ltd, Tokyo). The forward (fwd) and reverse (rev) primer sequences were as follows: *Sirt1* (fwd: 5^′^-TGCAGACGTGGTAATGTCCAAAC-3^′^; rev: 5^′^-ACATCTTGGCAGTATTTGTGGTGAA-3^′^), *GLUT4* (fwd: 5^′^-TTTCCAGCAGATCGGCTCTGACGA-3^′^; rev: 5^′^-TAGCCAAACTGAAGGGAGCCAAGC-3^′^), *ACO* (fwd: 5^′^-GGTTGTCATCGCTTTGGTGCCTGT-3^′^; rev: 5^′^-TAACTCTGGATTGAAGGTGGCGGCG-3^′^), *FATP-1* (fwd: 5^′^-CGGTGTGGTGGCTGCTCTTCTCAA-3^′^; rev: 5^′^-CGCTGCCATCTCCCCGCCATAAAT-3^′^), *GAPDH* (fwd: 5^′^-CAAGGTCATCCATGACAACTTTG-3^′^; rev: 5^′^-GGCCATCCACAGTCTTCTGG-3^′^). The reaction mixture containing reverse-transcribed cDNAs was preheated for 10 min at 95°C to activate the Taq polymerase. Fifty cycles of PCR, each consisting of a 10-s denaturation step at 95°C, a 15-s annealing step at 60°C, and a 15-s extension step at 72°C, was then performed. Throughout real-time PCR analysis, product identities were confirmed by melting curve analysis. The quantification of each gene was determined relative to a standard curve generated from a serially diluted sample. The ratios of the amounts of target mRNA to the amount of the internal standard (*GAPDH*) mRNA was determined as an arbitrary unit.

### Western blotting

Whole cell lysates for western blotting were harvested in RIPA buffer (Wako Pure Chemical Industries, Ltd., Tokyo) containing protease inhibitors (Takara Bio Inc., Shiga) and phosphatase inhibitor (Nacalai Tesque, Kyoto, Japan). Protein concentrations in the supernatants were determined using the Bio-Rad protein assay (Bio-Rad Laboratories, Inc., Tokyo). Twenty micrograms of protein were separated on a 5–20% gradient SDS-PAGE gel (ATTO Corporation, Tokyo) and transferred to polyvinylidene difluoride membranes (Millipore Corp., Billerica, MA). Membranes were blocked for 1 h at 20-25°C with 2% bovine serum albumin (BSA) or 5% Skim milk tris buffer saline Tween 20 (TBST) buffer. The membranes were incubated with appropriate dilutions of the primary antibodies (Toyobo Co., Ltd., Tokyo), viz., anti-Adiponectin (PA1-054 [1:2500 dilution]; Thermo Fisher Scientific Inc., Rockford, Illinois), anti-Phospho-AMPKα (Thr172) (2531 [1:5000 dilution]; Cell Signaling Technology, Inc., Beverly, MA), anti-AMPKα (2532 [1:5000 dilution]; Cell Signaling Technology, Inc.), and anti-GAPDH (2118 [1:5000 dilution]; Cell Signaling Technology, Inc.), overnight at 4°C. After washing, the blots were then incubated with HRP-conjugated anti-rabbit secondary antibody (7074 [dilution 1:5000], Cell Signaling Technology, Inc.) for 1 h in a room with a temperature maintained at 22 ± 2°C. Signals were detected using the Amersham™ *ECL*™ *Prime* enhanced chemiluminescence system (GE Healthcare Japan, Tokyo) and LAS-3000 mini imaging system (FujiFilm, Tokyo).

### Statistical analysis

All data are expressed as the means ± standard error (SE). Statistical analysis was analyzed with Student’s *t*-test for parametric comparison between 2 groups, or analysis of variance (ANOVA) with post-hoc testing by Fisher’s protected least significant difference (PLSD) test for multiple comparisons. Values of p < 0.05 were considered statistically significant.

## Results

### *In vivo* study

**Figure 1:**
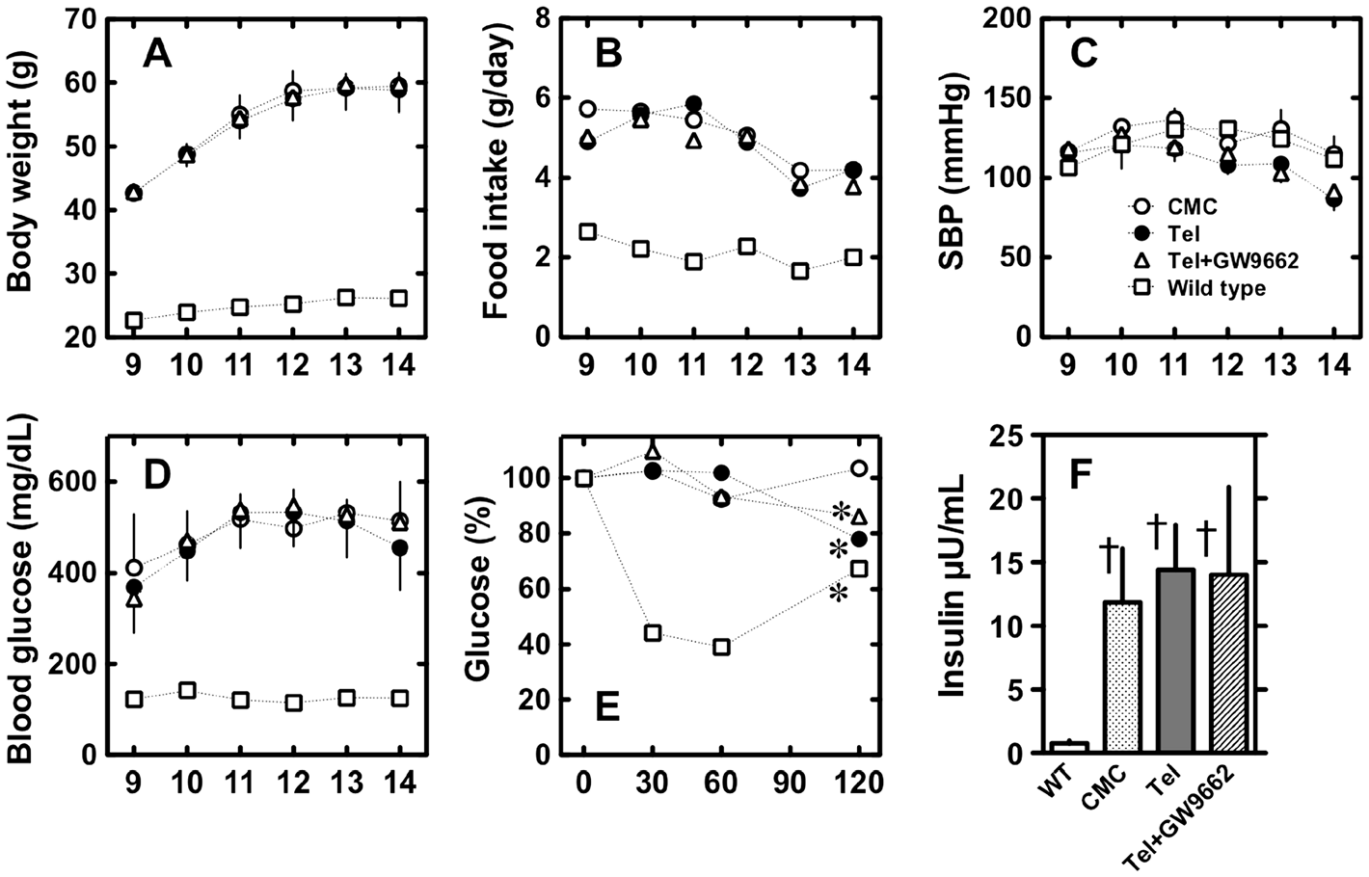
**Effects of telmisartan on *db/db *mice fed a high-fat diet.** In *db/db* mice fed a high-fat diet with CMC (○, n = 6), telmisartan (·, n = 6), or telmisartan + GW9662 (△, n = 7) for 5 weeks, body weight (**A**), food intake (**B**), systolic blood pressure (**C**), blood glucose (**D**), glucose levels during the insulin tolerance test (**E**), and fasting insulin levels (**F**) were measured. Values for wild type mice fed normal chow (□, n = 7) are also shown for reference. Glucose levels during the insulin tolerance test were measured after intraperitoneal injection with 0.75 U/kg insulin. Values shown represent means ± S.E. *p < 0.05 vs. 0 min and †p < wild type, by Fisher’s PLSD.

During the 5 weeks of the study period, *db/db* mice fed a high-fat diet gained weight, irrespective of whether they were treated with CMC, telmisartan, or telmisartan + GW9662 (Figures 1A and B). Systolic blood pressure remained unchanged in all groups during the study period (Figure 1C). Overnight fasting glucose levels were higher in the *db/db* mice than in the wild-type mice, but were not different among the 3 treatment *db/db* groups (Figure 1D). Plasma levels of adiponectin were not significantly different among groups [wild-type 5.22 ± 0.49 μg/mL (n = 6), CMC 9.14 ± 2.88 (n = 5) telmisartan 9.09 ± 1.07 (n = 4), and telmisartan + GW9662 6.67 ± 0.49 (n = 4)].

The ITT at the end of the 5-week study period demonstrated that the 120-min glucose levels were significantly decreased in telmisartan-treated and telmisartan + GW9662-treated mice as compared to CMC-treated mice (Figure 1E). The baseline insulin levels, measured before the ITT, had been comparable among the 3 *db/db* groups (Figure 1F).

### Effects of telmisartan on *Sirt1*, *PGC1α*, and *GLUT4* mRNA expression

**Figure 2:**
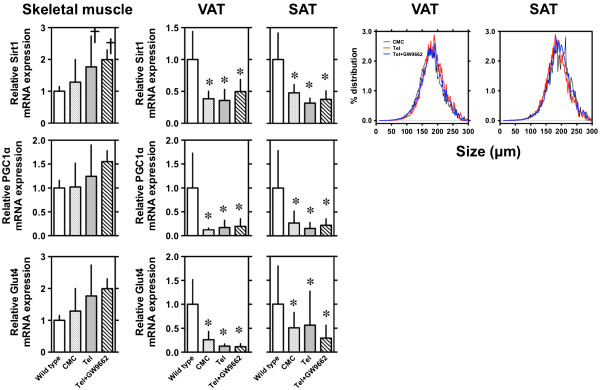
**Effects of telmisartan on mRNA expression and adipocyte size distribution in *db/db *mice fed a high-fat diet.** At sacrificed after 5-week treatment, *Sirt1*, *PGC1α*, and *GLUT4* mRNA expression were semi-quantified by RT-PCR in skeletal muscle and in visceral adipose tissue (VAT) and subcutaneous adipose tissue (SAT). GAPDH was used as an internal control. Distribution of adipocyte size was determined by the Coulter Multisizer III (Beckman Coulter, High Wycombe, England) after fixed with 2% osmium tetroxide. Values shown represent means ± S.E (n = 3-4). *p < 0.05 vs. wild type and †p < 0.05 vs. CMC by Fisher’s PLSD.

In skeletal muscle, expression of *Sirt1* mRNA was increased in telmisartan-treated mice compared to CMC-treated mice, but the expression was similar in telmisartan- and telmisartan + GW9662-treated mice (Figure 2). Expression of *PGC1α* and *GLUT4* mRNA was not different between CMC-treated and telmisartan-treated mice.

In visceral adipose tissue (VAT) and subcutaneous adipose tissue (SAT), expression of *Sirt1*, *PGC1α*, and *GLUT4* mRNA were profoundly decreased in CMC-treated *db/db* mice as compared to wild type. The expression was comparable between telmisartan-treated and telmisartan + GW9662-treated mice.

### Effects of telmisartan on the size of adipocytes and pancreatic islets in *db/db* mice fed a high-fat diet

**Figure 3:**
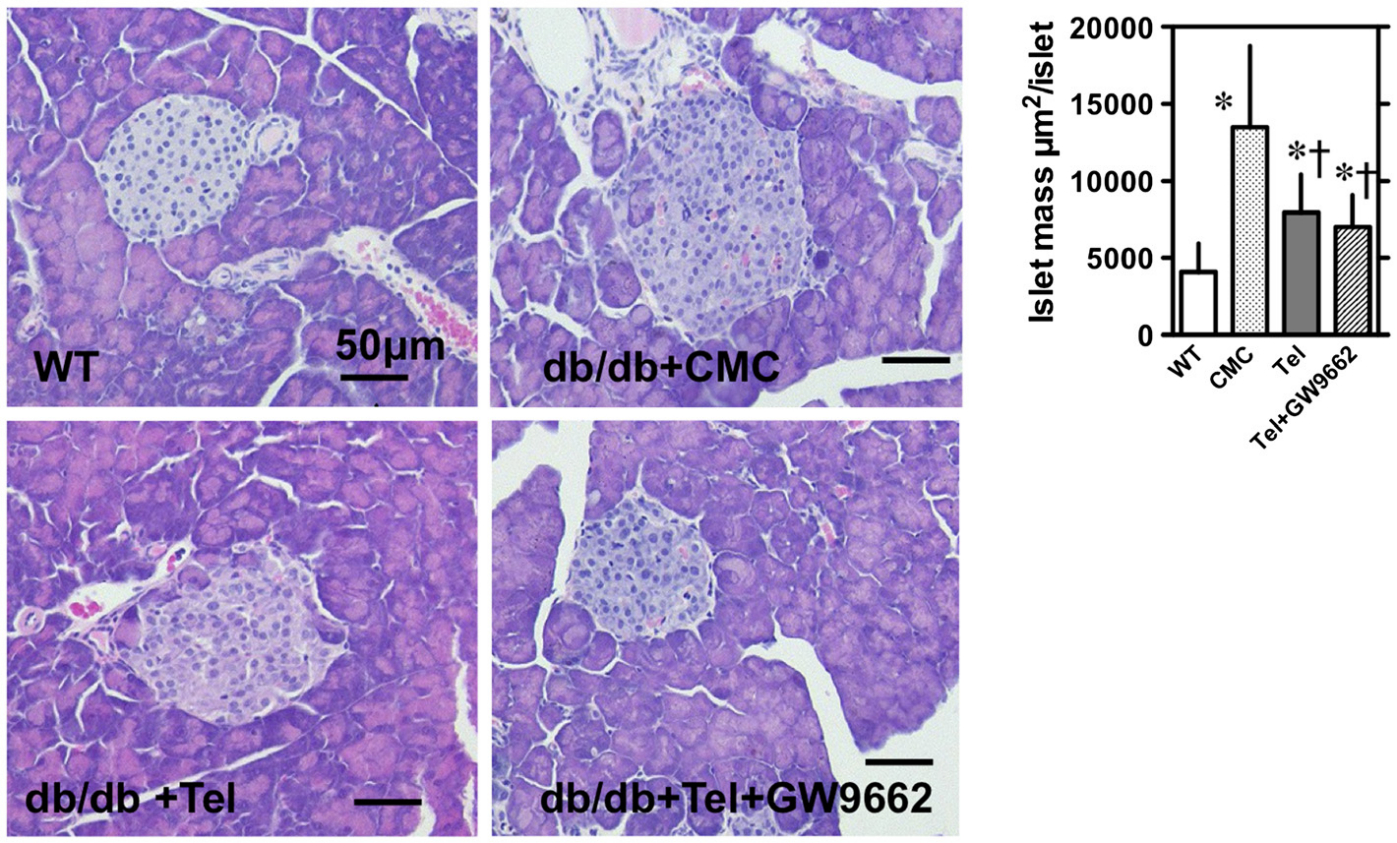
**Effects of telmisartan on the morphology and mass of pancreatic islets in *db/db *mice fed a high-fat diet.** Representative sections of paraffin-embedded serial sections of pancreata (5-μm m thickness) in wild type mice or *db/db* mice fed a high-fat diet with CMC, telmisartan, or telmisartan + GW9662 were stained for hematoxylin and eosin (HE). The size of pancreatic islets was determined from manually traced islet area. Values shown represent means ± S.E (n = 115-150). †p < 0.05 vs. CMC by Fisher’s PLSD.

The mean size of adipocyte in VAT and SAT was ≈ 2 times larger in db/db mice as compared to wild type (mean size of VAT 88 μm and SAT 94 μm). There were no significant differences in the size distribution (Figure 2) and the mean size among CMC-, telmisartan-, and telmisartan + GW9662-treated mice: 171 μm, 179 μm, and 182 μm in VAT and 181 μm, 180 μm, and 178 μm in SAT. However, although pancreatic islet size was increased in CMC-treated compared to wild-type mice, it was significantly decreased in telmisartan- and telmisartan + GW9662-treated mice (Figure 3).

### Effect of telmisartan on AMPK-SIRT1 pathway

**Figure 4:**
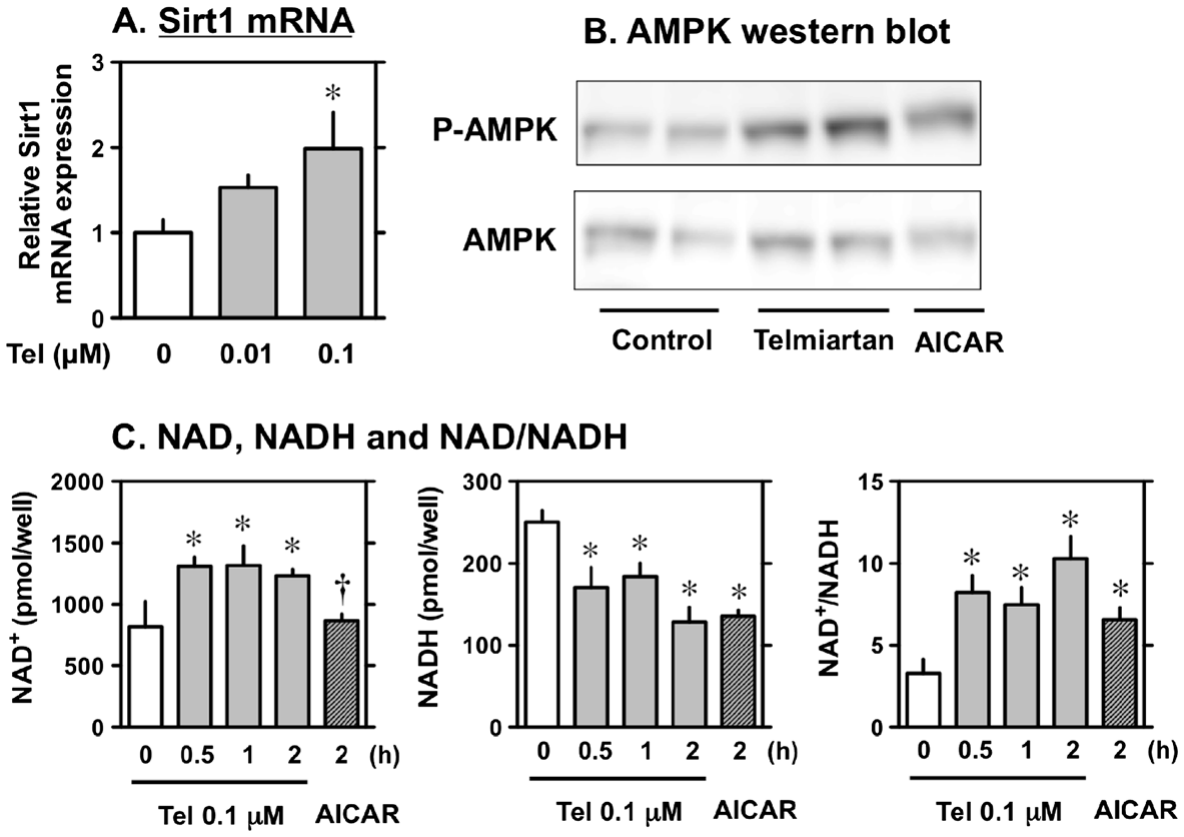
**Effects of telmisartan on the AMPK/SIRT1 pathway in C2C12 myocytes.** C2C12 myocytes were treated with vehicle or 0.01–0.1 μM telmisartan and the level of Sirt1 mRNA measured by RT-PCR (**A**). On day 5, C2C12 myocytes were treated with 0.1 μM telmisartan or 2 mM 5-aminoimidazole-4-carboxamide ribonucleoside (AICAR), an activator of AMP-activated protein kinase (AMPK), for 60 min, after which total cell lysates were subjected to western blot analysis using antibodies specific for Phospho-AMPK and total AMPK (**B**). NAD^+^, NADH content, and NAD^+^/NADH ratio (**C**) in C2C12 myocytes treated with telmisartan or AICAR (0.5 mM). Values shown represent means ± S.E (n = 3-5). *p < 0.05 vs. 0, by Fisher’s PLSD.

Since telmisartan increased *Sirt1* mRNA levels in the skeletal muscle of *db/db* mice, we investigated whether telmisartan administration could directly affect *SIRT1* signals in C2C12 myoblasts. Treatment with telmisartan increased *Sirt1* mRNA in C2C12 cells (Figure 4A). Treatment of C2C12 cells with telmisartan resulted in activation of AMPK, by means of phosphorylation, to a level comparable to that induced by 5-aminoimidazole-4-carboxamide ribonucleoside (AICAR), an activator of AMPK (Figure 4B). Treatment of C2C12 cells with telmisartan increased NAD^+^ and decreased NADH, resulting in an increase in the NAD^+^/NADH ratio, to a level comparable to that induced by AICAR (Figure 4C).

### Effect of telmisartan on mRNA expression of AMPK/SIRT1 pathway-related molecules in C2C12 myocytes

**Figure 5:**
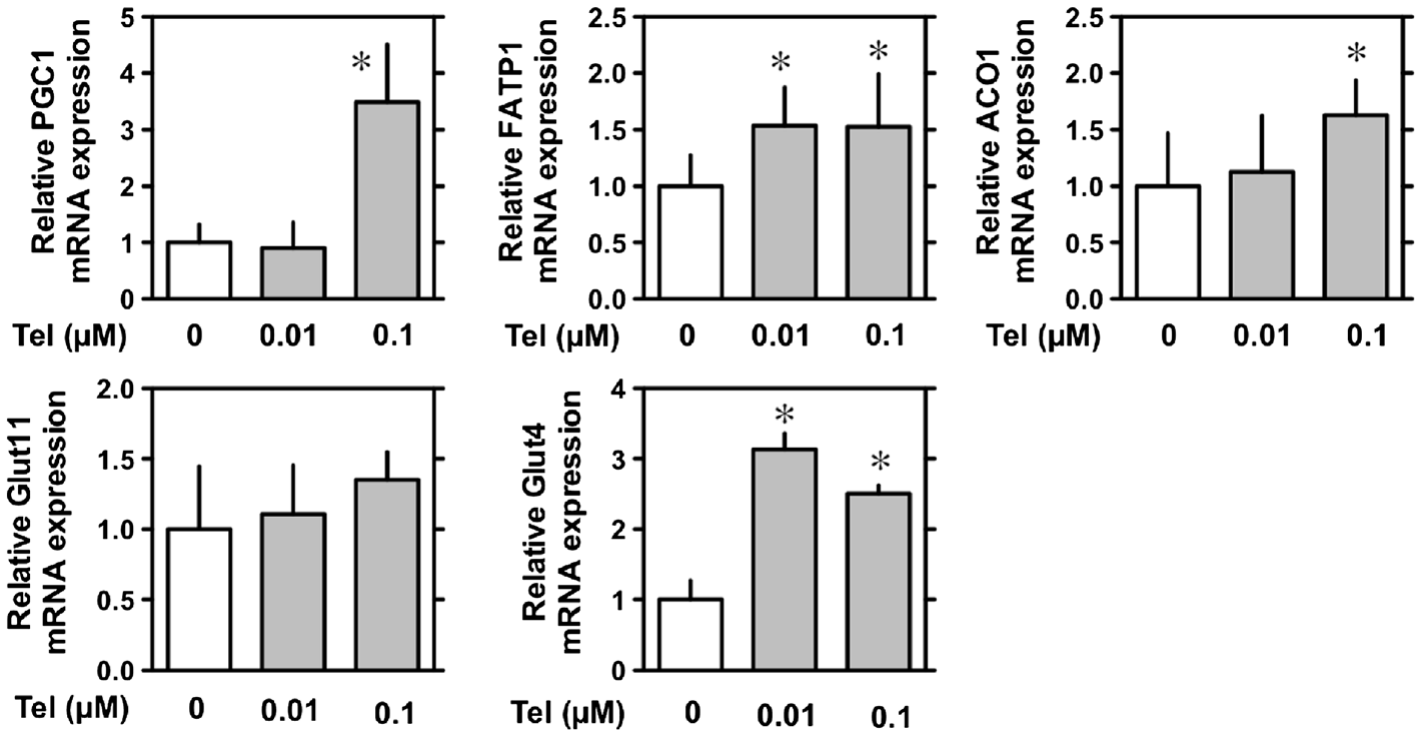
**Effects of telmisartan on AMPK/SIRT1 pathway in C2C12 myocytes.** On day 5, C2C12 myocytes were treated with telmisartan (indicated concentrations) for 12 h and analyzed by semi-quantitative RT-PCR. GAPDH was used as an internal control. Values shown represent the means ± S.E (n = 3-5). *p < 0.05 vs. 0 μM or baseline, by Fisher’s PLSD.

Treatment with telmisartan increased the mRNA levels of *PGC1*, *FATP1*, *ACO* and *GLUT4*, but not of *GLUT1*, in C2C12 myocytes (Figure 5).

## Discussion

Here, we showed that telmisartan treatment improved insulin sensitivity in obese *db/db* mice fed a high-fat diet and led to reduction in the size of hypertrophic pancreatic islets in these mice. Moreover, we demonstrated that *in vitro* treatment with telmisartan led to increased expression of *Sirt1* mRNA in skeletal muscle cells; the increase in *Sirt1* mRNA in telmisartan-treated C2C12 myoblasts occurred concomitantly with an increase in AMPK phosphorylation, an increase in NAD^+^/NADH ratio, and increases in the mRNA levels of *PGC1*, *FATP1*, *ACO*, and *GLUT4*.

It has previously been shown that levels of *Sirt1* mRNA and SIRT1 activity are decreased in an animal obesity model [25,26] and in obese humans [27]. To investigate the potential consequences of decreased SIRT1, we utilized *db/db* mice, a genetic model of obesity and insulin resistance resulting from a leptin receptor mutation [28]. On a high-fat diet, this *db/db* strain demonstrates severe insulin resistance and hyperglycemia [29]. In the current study, all mice fed a high-fat diet gained body weigh comparably (Figures 1A and B) and casual glucose levels did not differ among the 3 treatment groups (Figure 1D), but ITT-determined glucose levels were significantly decreased in telmisartan-treated mice. This indicates that administration of telmisartan ameliorates insulin resistance in the obesity and insulin resistance model. Because co-administration of GW9662, a selective PPARγ inhibitor, could not reverse this *in vivo* effect of telmisartan, the effects of telmisartan appear to act via a PPARγ-independent pathway. It has been suggested that some of angiotensin II receptor blockers (ARBs) have beneficial effects on lipid metabolism [30] and insulin sensitivity possibly through PPAR-γ activation [9-13]. We confirmed that 0.5-10 μM of telmisartan, but not up to 10 μM of olmesartan nor valsartan, have a PPAR-γ agonistic effect in HEK293 cells (data not shown). It should be clarified how different ARBs show mechanism(s) beyond blockade of renin-angiotensin system (RAS) in future studies.

Since it has been reported that AMPK activity and SIRT1 expression are decreased in the white adipose tissue (WAT) of *db/db* and HFD mice [26], we evaluated mRNA levels of *Sirt1*, *PGC1α*, and *GLUT4* in the VAT and SAT of the various *db/db* mouse treatment groups. Consistent with a previous study [26], vehicle-treated *db/db* mice showed a decreased expression of *Sirt1*, *PGC1α*, and *GLUT4* mRNAs in the VAT and SAT. Moreover, treatment with telmisartan did not change these expression levels, suggesting that telmisartan improved insulin resistance via a non-adipose tissue metabolic network in this *db/db* model. This hypothesis is supported by the finding that the adipocyte size distribution was not changed by telmisartan treatment. However, telmisartan administration did upregulate expression of *Sirt1* mRNA and levels of phospho-Thr172-AMPK in skeletal muscle.

Metabolic sensors such as AMPK and SIRT1 are the gatekeepers of the activity of the master regulator, the mitochondrion, and are crucial to the regulatory network for metabolic homeostasis [1,31,32]. Mice models mildly overexpressing SIRT1 demonstrated ameliorated glucose tolerance when insulin resistance and/or diabetes were induced [33,34]. Similarly, treatment with different SIRT1 agonists prevents weight gain and insulin resistance when mice were challenged with high-fat diets [8,35]. Similarly, pharmacological activation of AMPK by metformin [6] and A-769662 [7] ameliorated insulin resistance in animals fed a high-fat diet. These results indicate a role for AMPK/SIRT1 in the control of metabolic homeostasis. It is believed that metformin treatment is the successful approach to reduce insulin resistance mainly through activation of liver AMPK [6], whereas DPP-4 inhibitors exhibit the best effects after oral glucose loading, emphasizing the incretin effect after oral stimulation [36]. However, metformin and DPP-4 inhibitors may involve different mechanisms in the treatment of mice fed high-fat diet [37]. Bourron et al. suggested a possible role of metformin on AMPK dependent lipolysis in adipocytes that may lead to lower plasma levels of fatty acids and to alleviating insulin resistance [38]. Sitagliptin also decreased adipocyte size efficiently and, even though the precise mechanisms remain to be elucidated, DPP-4 inhibition produced extra-pancreatic effects, such as enhanced postprandial lipid mobilization and oxidation [36]. Altogether, upregulation of *Sirt1* mRNA and activation of AMPK in the skeletal muscle are associated with the improved insulin sensitivity observed upon telmisartan treatment.

*PGC1α* and *GLUT4* mRNA expression also tended to be increased in skeletal muscle cells. AMPK and SIRT1 have both been described to directly affect PGC-1α activity through phosphorylation and deacetylation, respectively [32]. Thus, we next evaluated the direct effect of telmisartan on PGC-1α activity in muscle using C2C12 mouse myoblast cells. We found that *in vitro* treatment with telmisartan increased *Sirt1* mRNA concomitantly with the increase in AMPK phosphorylation, an increase in the NAD^+^/NADH ratio, and the mRNA levels of *PGC1*, *FATP1*, *ACO*, and *GLUT4* in C2C12 cells. To our knowledge, the current study, for the first time, showed that treatment with telmisartan enhanced *Sirt1* mRNA and protein in the skeletal muscle.

SIRT1, an NAD^+^-dependent regulator of energy metabolism, has been reported to be activated through AMPK-mediated induction of nicotinamide phosphoribosyltransferase (NAMPT), the rate-limiting enzyme for NAD^+^ biosynthesis [1,8]. We thus examined the direct effects of telmisartan on AMPK. Telmisartan resulted in a significant increase in AMPKα phosphorylation at Thr172, to a level comparable to that induced by AICAR, an activator of AMPK.

**Figure 6:**
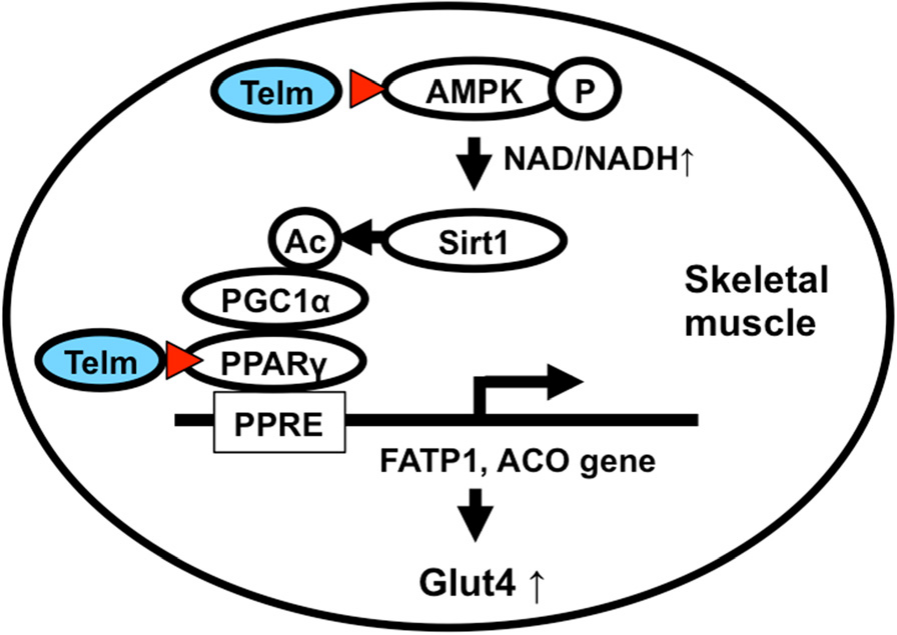
**A model of the proposed mechanism by which telmisartan improves insulin sensitivity in obese *db/db *mice.** Telm: telmisartan; ▲: site of action; Ac: acetylated; P: phosphorylated; PGC1α: peroxisome proliferator-activated receptor-γ coactivator-1α; PPRE: PPAR response element.

Taken altogether, we propose a possible mechanism by which telmisartan improves insulin sensitivity in skeletal muscle in Figure 6D. Impaired adipocytokine signaling, caused by suppression of expression of leptin and adipoR2 receptors, could lead to decreased activation of AMPK. This, in turn, may result in decreased NAMPT transcription, and consequently decreased SIRT1 activity and expression. Our results are also consistent with the notion that telmisartan, besides blocking AT1, acts to promote adipocyte differentiation and improve insulin sensitivity.

As previously reported, pancreatic islets in *db/db* mice on a high-fat diet were profoundly hypertrophic, presumably as compensation for severe insulin resistance. Saitoh et al. reported that telmisartan decreased the accumulation of palmitate-induced reactive oxygen species in MIN6 cells by 25%, and by 55% in mouse islet cells, suggesting that RAS blockade may be a possible mechanism for protecting β-cell survival and preserving insulin secretion capacity [39]. Yuan et al. reported that telmisartan and perindopril, a RAS inhibitor, comparably improved islet morphology, and significantly reduced *IL-1β*, *HIF-1α,* and *CHOP* mRNA in Wistar rats fed a high-fat diet for 16 weeks [40]. They suggested that blockade of RAS may protect the islet function of rats on a long-term high-fat diet, via down-regulation of islet inflammation, oxidative stress, endoplasmic reticulum stress, and apoptosis [41]. Our study showed that such hypertrophic pancreatic islets were improved in telmisartan-treated, and in telmisartan + GW9662-treated mice. The distribution in pancreatic β cell mass may also be affected by telmisartan, but we could not determine variation in the β cell mass distribution by our method. Although we could not determine the mechanism by which telmisartan affects islet morphology and function, future studies should determine the precise mechanism of the antimetabolic effects of this drug.

Our study had some limitations. First, the C2C12 model has limitations in comparison to freshly prepared frozen skeletal muscle cells [23]: C2C12 cells were originally obtained by Yaffe and Saxel through serial passage of myoblasts cultured from the thigh muscle of C3H mice after a crush injury. C2C12 cells are a useful tool for studying the differentiation of myoblasts and osteoblasts, expressing various proteins, and exploring mechanistic pathways. Nevertheless, the C2C12 cell line originated from a single clone, and thus has clone-specific traits, and fails to recapitulate primary cells. Second, the use of a single concentration of GW9662 may not be specific for verifying inhibition of PPARγ activity. We used a single dose of 1 mg/kg of GW9662 for the *in vivo* studies, and a single dose of 5 μM of GW9662 for the *in vitro* studies. We determined the effect of telmisartan on the activity of a PPARγ2 promoter luciferase gene reporter construct, indicating that 0.5–10.1 mM of telmisartan increased the activity of the *PPARγ2* gene promoter by 1.8- to 2-fold, to a ≃30% level comparable to that achieved by pioglitazone, a known PPARγ agonist (in submission). The effects of telmisartan through PPARγ-dependent and -independent pathways need to be confirmed in further studies. Third, nonspecific effects of the drugs used in this study cannot be eliminated. GW9662 has been shown to inhibit COX-2 activity in addition to having an inhibitory effect on PPARγ, the active derivative of AICAR modulates other AMP-sensitive enzymes, and the selectivity of compound C for inhibition of AMPK remains uncertain. The findings presented in this study should be confirmed by using dominant-negative vectors or siRNA knockdown of AMPK and SIRT1 in the future. Fourth, our study could not determine whether telmisartan regulates AMPK/SIRT1 pathways in a tissue- or model-specific manner. Caton et al. reported that AMPK activity, NAMPT expression and SIRT1 expression were decreased in the WAT of *db/db* and HFD mice [26]. In addition, they showed that metformin increased the AMPK activity in the WAT of *db/db* mice and in adipocytes treated with metformin, with increased NAMPT and SIRT1 levels. In our study, telmisartan showed significant changes in protein and mRNA levels in skeletal muscle, but not in WAT. Although we cannot explain this discrepancy between the 2 studies, Chen et al. reported that SIRT1 expression is differentially regulated, in a tissue-specific manner upon calorie restriction, which may reflect tissue-specific roles of SIRT1 [41]. Tissue-specific effects of telmisartan need to be elucidated in future studies. Finally, the estimation of cell size has a limitation: in the fat pad, central adipocytes are usually bigger than peripheral adipocytes, and islets are roughly spheres and sections could cut spheres at its poles and distort the results.

In conclusion, telmisartan treatment improved glucose intolerance in *db/db* mice fed a high-fat diet, which was not inhibited by GW9662 co-administration. Telmisartan increased *Sirt1* mRNA concomitantly with activation of AMPK via phosphorylation, an increase in NAD^+^/NADH ratio, and increase in mRNA levels of *PGC1α*, *FATP1*, *ACO*, and *GLUT4* in C2C12 cell lines, suggesting that these improvements were at least partially due to the direct actions of telmisartan on AMPK/SIRT1 pathways. Precise understanding of this molecular mechanism will require further investigations.

## Abbreviations

ANOVA: Analysis of variance; AT1: Angiotensin II type 1 receptor; DMEM: Dulbecco’s modified Eagle’s medium; DMSO: Dimethyl sulfoxide; G6PDH: Glucose-6-phosphate dehydrogenase; ITT: Insulin tolerance test; NAD: Nicotinamide adenine dinucleotide; PPAR: Peroxisome proliferator-activated receptor; RT-PCR: Real-time-polymerase chain reaction; Sirt1: Nicotinamide adenine dinucleotide (NAD)-dependent deacetylase.

## Competing interests

The authors declare that they have no competing interests.

## Authors' contributions

AS, MS, MS: design and conducting the study, data collection, analysis, and manuscript writing; HA, MH, YH, HK, YN, HM: contribution to design and discussion; HA and HA: contribution to cell distribution study; NM and IS: contribution to C2C12 study. We sincerely thank Mss. ST, JK and HG for her assistance. All authors read and approved the final manuscript.
